# Characteristics of Study Design and Statistical Analysis in OCT-Based Studies of Neurodegeneration

**DOI:** 10.1016/j.xops.2025.100961

**Published:** 2025-10-08

**Authors:** Sonny Caplash, Helen Song, Amy Waldman, Gui-Shuang Ying, Benjamin J. Kim

**Affiliations:** 1 Department of Ophthalmology, Scheie Eye Institute, University of Pennsylvania, Philadelphia, Pennsylvania; 2 Perelman School of Medicine, University of Pennsylvania, Philadelphia, Pennsylvania; 3 Department of Neurology, Children’s Hospital of Philadelphia, University of Pennsylvania, Philadelphia, Pennsylvania; 4 Department of Ophthalmology, Center for Preventive Ophthalmology and Biostatistics, University of Pennsylvania, Philadelphia, Pennsylvania

**Keywords:** Alzheimer disease, Multiple sclerosis, Neurodegenerative diseases, Optical coherence tomography, Retina

## Abstract

**Purpose:**

To evaluate the design and statistical analysis of recently published clinical studies of retina OCT of neurodegenerative diseases.

**Design:**

Review of 134 publications.

**Methods:**

Clinical and translational publications from 2013-2023 involving human OCT studies were reviewed. Publications were restricted to the top 10 journals of either ophthalmology or neurology based on the SCImago Journal Rank. Data were extracted and analyzed regarding study design characteristics, disease studied, imaging characteristics, and statistical analysis method.

**Main Outcome Measures:**

Characteristics of study design and statistical analysis.

**Results:**

Of the 134 studies, the most commonly investigated diseases were multiple sclerosis (47.8%), Alzheimer disease (31.3%), and Parkinson disease (14.2%). One hundred three (76.9%) studies were cross-sectional, and 31 (23.1%) studies were longitudinal. Of the 42 Alzheimer disease studies, 19.0% used cerebrospinal fluid biomarkers, 26.2% used positron emission tomography imaging, and 4.8% used genetic data to confirm the diagnosis. The peripapillary retinal nerve fiber layer thickness (95 studies) and macular retina layer thickness (96 studies) were the most commonly studied OCT measurements; 69 studies looked at both of these measurements. For 25 (18.7%) of the studies, no statement of image quality criteria or exclusion of subjects or eyes because of image quality criteria was identified. One hundred seventeen (87.3%) studies performed bilateral eye imaging, but only 89 (66.4%) studies performed bilateral image analysis. Of the 89 studies with bilateral eye image analysis, 53 (59.6%) performed analysis at the eye level by combining data from the left and right eye, making each eye (rather than each subject) the unit of analysis. Of the 53 studies performing analysis at the eye level for data from both eyes, the intereye correlation was adjusted in 38 (71.7%) studies, while 15 (28.3%) studies did not account for the intereye correlation. Thirty-seven (27.6%) studies corrected for multiple comparisons.

**Conclusion:**

There may be opportunities for improvement in the study design and statistical analysis of OCT studies of neurodegenerative diseases.

**Financial Disclosure(s):**

Proprietary or commercial disclosure may be found in the Footnotes and Disclosures at the end of this article.

Neurodegenerative disorders are a wide spectrum of disorders and include diseases such as Alzheimer, Parkinson, frontotemporal dementia, and multiple sclerosis. They impact a substantial degree of an increasingly older population. It has been estimated that roughly 6.9 million people aged ≥65 years have Alzheimer disease in the United States.^1^ Globally, the prevalence of Parkinson disease has increased to >8.5 million in 2019.^2^ As the global population survives further into older age, each of these diseases and the broader diagnosis of dementia are projected to increase in prevalence.^3^, ^4^, ^5^, ^6^ Multiple sclerosis is a nonage related neurodegenerative disorder driven by chronic inflammation. Early in the disease course are hallmark autoimmune flares, and there is also progressive neurodegeneration that is most evident in later stages of multiple sclerosis.^7^ Multiple sclerosis has a significant impact on the global population with an estimated prevalence of roughly 2.8 million in 2020.^8^ The global burden of these diseases underscores the need for effective diagnosis, prognostication, and therapeutic investigations.

A growing body of evidence has linked retinal OCT measurements with neurodegenerative disorders.^9^, ^10^, ^11^, ^12^, ^13^, ^14^ The basis of the connection between OCT measurements and neurodegeneration centers around the retina’s embryologic and physiologic connection with cortical brain matter. Specifically, the embryologic origin of the human retina stems from the optic vesicle, an outpouching of the early human forebrain.^15^, ^16^, ^17^ Connections between cortical pathology and retinal cells have been implicated by histopathologic studies.^18^, ^19^, ^20^, ^21^ Multiple studies have suggested the potential utility of OCT as a low-cost and noninvasive biomarker for the diagnosis and clinical care of patients with neurologic disorders.^9^, ^10^, ^11^, ^12^, ^13^, ^14^

The increasing number of clinical studies assessing the relationship between OCT measurements and neurodegenerative disorders brings their methodology under scrutiny. For example, ophthalmic measurements are unique in that they are commonly obtained from both eyes of each subject, and the data from 2 eyes of a subject are positively correlated, though not all clinical studies consider this in their study design and statistical analysis.^22^, ^23^, ^24^ Excluding the second eye in statistical analysis can decrease the volume of information analyzed, causing the statistical power to be suboptimal. Conversely, incorporating second eye data without appropriately correcting for intereye correlation can result in faulty statistical analysis and invalid conclusions.^22^ Further, the methods of image analysis and the criteria for diagnosis, particularly for dementia studies, deserve review. The margin of change in OCT measurements between controls and those with neurocognitive disorders is often on the order of microns,^9^^,^^10^^,^^25^^,^^26^ and even the smallest biases from suboptimal analysis can impact the clinical validity of results.

The purpose of this study is to evaluate the features of study design and statistical analysis methods in clinical studies investigating the association of potential OCT biomarkers and neurodegenerative disorders. This study also aims to provide context for interpretation of current data and guide optimal study design and statistical analyses for future studies.

## Methods

A comprehensive literature search was performed by pairing “optical coherence tomography” with several specific keywords: “Alzheimer’s,” “dementia,” “mild cognitive impairment,” “multiple sclerosis,” and “Parkinson’s.” Results were refined to a specific timeframe of July 2013 to July 2023. Next, the SCImago Journal Rank was used to further restrict results by the top 10 journals in each specialty (ophthalmology and neurology; Table 1). Among all studies that satisfied the aforementioned criteria, results were further narrowed down based on study design. Specifically, only translational and clinical studies using OCT imaging in human subjects were included, and studies involving cadaver-based OCT applications were excluded.

**Table 1:** Journals, Year of Publication, and Corresponding Author Characteristics for Included Papers (N = 134)

Journals	
Invest Ophthalmol Vis Sci	18 (13.43%)
Neurology	18 (13.43%)
Acta Ophthalmol	12 (8.96%)
Alzheimers Res Ther	11 (8.21%)
Ann Neurol	10 (7.46%)
Br J Ophthalmol	8 (5.97%)
Brain	8 (5.97%)
Eye (Lond)	8 (5.97%)
JAMA Neurol	8 (5.97%)
JAMA Ophthalmol	7 (5.22%)
Ophthalmology	7 (5.22%)
J Neurol Neurosurg Psychiatry	4 (2.99%)
Am J Ophthalmol	3 (2.24%)
Retina	3 (2.24%)
Eye Vis (Lond)	2 (1.49%)
Ophthalmol Retina	2 (1.49%)
Acta Neuropathol Commun	2 (1.49%)
Alzheimers Dement	1 (0.75%)
Lancet Neurol	1 (0.75%)
Mov Disord	1 (0.75%)
Year of publication	
2013	3 (2.24%)
2014	17 (12.69%)
2015	7 (5.22%)
2016	9 (6.72%)
2017	11 (8.21%)
2018	15 (11.19%)
2019	12 (8.96%)
2020	19 (14.18%)
2021	15 (11.19%)
2022	15 (11.19%)
2023	11 (8.21%)
Country of the corresponding author (ISO country code)	
USA	42 (31.34%)
ESP	27 (20.15%)
NLD	9 (6.72%)
KOR	8 (5.97%)
ITA	7 (5.22%)
CHN	6 (4.48%)
DEU	6 (4.48%)
GBR	5 (3.73%)
TUR	5 (3.73%)
AUS	3 (2.24%)
AUT	3 (2.24%)
FRA	3 (2.24%)
ISR	2 (1.49%)
SGP	2 (1.49%)
BEL	1 (0.75%)
CHE	1 (0.75%)
JPN	1 (0.75%)
NZL	1 (0.75%)
POL	1 (0.75%)
QAT	1 (0.75%)
Specialty of the corresponding author(s)	
Ophthalmology	62 (46.27%)
Neurology	51 (38.06%)
Other	12 (8.96%)
Neurology and ophthalmology	5 (3.74%)
Ophthalmology and other	4 (2.99%)

AUS = Australia; AUT = Austria; BEL = Belgium; CHE = Switzerland; CHN = China; DEU = Germany; ESP = Spain; FRA = France; GBR = Great Britain; ISO = International Organization for Standardization; ISR = Isreal; ITA = Italy; JPN = Japan; KOR = Korea; NLD = Netherlands; NZL = New Zealand; POL = Poland; QAT = Qatar; SGP = Singapore; TUR = Turkey; USA = United States of America.

Once all eligible studies were identified by author H.S., they were confirmed by author B.J.K. After confirmation of eligible studies, data for the features of study design and statistical analysis were extracted from each study by author H.S. (Table 1, Table 2, Table 3) including:

1. General paper characteristics: whether the journal or corresponding author was in the specialty of ophthalmology versus neurology, country of corresponding author, year of publication, study type (cross sectional vs. longitudinal).
2. Disease of interest and whether biomarker testing was done to confirm clinical diagnosis for studies of dementia (eg, cerebrospinal fluid [CSF] biomarkers for Alzheimer disease).
3. Imaging characteristics: type of imaging, measurement, and method (eg, spectral-domain OCT of macula retinal layers with Heidelberg Explorer auto-measurement), and whether image quality criteria were stated in study methodology. It was also determined whether the following was performed: manual adjustment of image measures, adjustment of measures based on refraction or axial length, and obtaining images from both eyes.
4. Statistical analysis characteristics: unilateral versus bilateral eye image analysis, intereye correlation adjustment or other method of data statistical analysis for bilateral cases, *P* value correction for multiple comparisons, and inclusion of a power calculation.

**Table 2:** Characteristics of Studies

Disease of Interest (N = 134)	
Multiple sclerosis	64 (47.76%)
Alzheimer disease	42 (31.34%)
Parkinson disease	19 (14.18%)
>1 disease	5 (3.73%)
Unspecified dementia	2 (1.49%)
Frontotemporal dementia	2 (1.49%)
OCT study type (N = 134)	
Cross-sectional	103 (76.87%)
Longitudinal	31 (23.1%)
Image type (N = 134)	
SD-OCT	96 (71.64%)
SD-OCT, SD-OCTA	12 (8.96%)
SD-OCT, other	10 (7.46%)
SD-OCTA	5 (3.73%)
SS-OCT, SS-OCTA	3 (2.24%)
SD-OCT, SD-OCTA, other	2 (1.49%)
SS-OCT	2 (1.49%)
SS-OCTA	2 (1.49%)
Polarization-sensitive OCT	1 (0.75%)
SLO-OCT	1 (0.75%)
With fundus photography (N = 134)	
No	105 (78.36%)
Yes	29 (21.64%)
Type of image measures (not mutually exclusive)∗	
pRNFL thickness	95 (35.45%)
Macular retinal layer thickness	96 (35.82%)
OCT angiography (FAZ, vessel density, perfusion)	25 (9.33%)
Macular retinal layer volume/total macular volume	25 (9.33%)
Choroidal thickness (3/9 with choroidal vascularity index)	9 (3.36%)
Other peripapillary measurements†	5 (1.87%)
Other‡	13 (4.85%)
OCT image measurement method (not mutually exclusive)∗	
Heidelberg auto-measurement	58 (29.00%)
Cirrus auto-measurement	34 (17.00%)
Other auto-measurement	31 (15.50%)
Multiple measurement algorithms	29 (14.50%)
Topcon auto-measurement	10 (5.00%)
Optovue auto-measurement	10 (5.00%)
Manual measurement	9 (4.50%)
Auto & manual measurements	7 (3.50%)
Iowa Reference Algorithm	4 (2.00%)
Not specified	8 (4.00%)
Manual measurement correction (N = 134)	
No	80 (59.70%)
Yes	40 (29.85%)
Not specified	9 (6.72%)
Not applicable	5 (3.73%)
Image quality criteria (N = 134)	
Yes	109 (81.34%)
No (no statement about exclusion of images with limited quality)	25 (18.66%)
Image quality criteria used (N = 134)	
OSCAR-IB	38 (28.36%)
Signal strength threshold	30 (22.39%)
Image quality score threshold	11 (8.21%)
Artifact/miscentering/segmentation error	5 (3.73%)
Signal:noise threshold	3 (2.24%)
OSCAR-IB, artifact/miscentering/segmentation error	1 (0.75%)
Signal strength threshold, image quality score threshold	2 (1.49%)
Signal:noise threshold, other	1 (0.75%)
Other	1 (0.75%)
Nonspecific statement about excluding images with limited quality	17 (12.69%)
Not applicable	25 (18.66%)
Refractive error threshold applied (N = 134)	
Yes	91 (67.91%)
No	43 (32.09%)
Spherical refractive error threshold (diopters)	
n	68
Mean (SD)	5.4 (0.8)
Median (Q1, Q3)	6.0 (5.0, 6.0)
Cylindrical refractive error threshold (diopters)	
n	28
Mean (SD)	2.9 (0.9)
Median (Q1, Q3)	3.0 (2.0, 3.0)
Refractive error or axial length correction (N = 134)	
No	122 (91.04%)
Yes	12 (8.96%)
Number of subjects analyzed	
n (papers)	131
Mean (SD)	1174.4 (7638.2)
Median (Q1, Q3)	113.0 (60.0, 212.0)
Min, max	5.0, 72 083.0
Data not clearly specified (papers)	3
Number of eyes analyzed	
n (papers)	108
Mean (SD)	2527.1 (16 392.8)
Median (Q1, Q3)	166.0 (83.0, 339.0)
Min, max	7.0, 144 166.0
Data not clearly specified (papers)	26
Images from both eyes acquired (N = 134)	
No	13 (9.70%)
Yes	117 (87.31%)
Not recorded, when a value is expected	4 (2.99%)

FAZ = foveal avascular zone; OSCAR-IB = (O) obvious problems, (S) poor signal strength, (C) centration of scan, (A) algorithm failure, (R) retinal pathology other than MS related, (I) illumination, and (B) beam placement; pRNFL = peripapillary retinal nerve fiber layer; SD = standard deviation; SD-OCT = spectral-domain OCT; SD-OCTA = spectral-domain OCT angiography; SLO-OCT = scanning laser ophthalmoscopy OCT; SS-OCT = swept-source OCT; SS-OCTA = swept-source OCT angiography.

∗ The total count can be >134 papers because each category is not mutually exclusive (ie, 1 paper can be in >1 category).

† Other peripapillary measurements: Axonal Nsite Analytics of RNFL and papillomacular bundle thickness (2); peripapillary inner nuclear layer, outer plexiform layer, and outer nuclear layer (1); Bruch membrane opening—minimum rim width (1); peripapillary ganglion cell layer (1).

‡ Other: nasal/temporal peripapillary bundle thickness ratio (1); peak systolic velocity, end diastolic velocity, resistivity index (1); RNFL thickness via scanning laser polarimetry (1); lamina cribrosa thickness (2); corneal nerve fiber density, and length (1); phase retardation per unit depth (1); fluorescence lifetime imaging ophthalmoscopy (1); number of hyperreflective features on adaptive optics (1); retinal blood flow via doppler OCT (1); relative reflectance on hyperspectral OCT (1); arterial and venous branch points on fundus arterial photography (1); adaptive optics classification of foveal structure (1).

**Table 3:** Characteristics of Statistical Analysis of Data

	Studies (%)
Statistical analysis for both eyes (N = 134)	
No	42 (31.34%)
Yes	89 (66.42%)
Not specified	3 (2.24%)
Selection criteria among studies using single eye (N = 42)∗	
Random	22 (52.38%)
Right eye	8 (19.04%)
Affected/unaffected eye	6 (14.29%)
Eye with best image quality	4 (9.52%)
Least symptomatic/best visual acuity	3 (7.14%)
Dominant eye	1 (2.38%)
Not recorded, when a value is expected	5 (11.90%)
Analysis approach among studies using both eyes (N = 89)∗	
Eye level analysis for both eyes	53 (59.55%)
Mean of eyes	18 (20.22%)
Intereye differences affected vs. unaffected eye	16 (17.98%)
Separate left & right analyses	10 (11.24%)
Intereye differences	9 (10.11%)
Mean of nonoptic nerve eyes	6 (6.74%)
Other	2 (2.25%)
Not recorded, when a value is expected	4 (4.49%)
Intereye correlation adjustment among studies that analyzed both eyes at eye level (N = 53)	
Yes	38 (71.70%)
No	15 (28.30%)
Adjusted method among studies that adjusted for intereye correlation (N = 38)	
Generalized estimating equations	21 (55.3%)
Mixed-effects model	13 (34.2%)
Other	4 (10.5%)
Correction for multiple measures (N = 134)	
No	62 (46.27%)
Yes	37 (27.61%)
Not applicable	35 (26.12%)
Method for correcting for multiple measures among those with multiple measure correction (N = 37)∗	
Bonferroni	26 (70.27%)
Holm–Bonferroni	3 (8.11%)
Benjamini–Hochberg	3 (8.11%)
False discovery rate	2 (5.41%)
Other	4 (10.81%)
Power calculation included (N = 134)	
No	119 (88.81%)
Yes	15 (11.19%)

∗ Total may be >100% because >1 method was used in the paper.

Descriptive analyses were performed to summarize the study characteristics using percentages, mean, median, and range. The chi-square test was used to evaluate factors associated with the adjustment of intereye correlation among studies that performed bilateral eye analyses. Two-sided *P* < 0.05 was considered significant.

## Results

One hundred thirty-four papers met all the inclusion criteria. All eligible journals were represented, and each of the years from 2013–2023 had papers meeting inclusion criteria (Table 1). Seventy-two (53.7%) of the 134 papers were published in the last 5 years of this 10-year period. Sixty-two (46.3%) of the corresponding authors were ophthalmologists, and 51 (38.1%) were neurologists. With regards to disease studied, the most common were multiple sclerosis (64 studies, 47.8%), Alzheimer disease (42 studies, 31.3%), and Parkinson disease (19 studies, 14.2%) (Table 2). For study design, 103 (76.9%) were cross-sectional, and 31 (23.1%) were longitudinal. The median follow-up in longitudinal studies was 36 months (range 12–120). Of the 31 longitudinal studies, the diseases investigated were multiple sclerosis (25 studies, 80.6%), Alzheimer disease (3 studies, 9.7%), and Parkinson disease (3 studies, 9.7%). Among the 42 studies of Alzheimer disease, 8 (19.0%) studies used CSF biomarkers, 11 (26.2%) studies used positron emission tomography (PET) imaging, and 2 (4.8%) studies used genetic data to confirm the diagnosis.

With regards to the specific imaging modality used, spectral-domain OCT was the most common (Table 2). The most common OCT measurements were peripapillary retinal nerve fiber layer thickness (95 studies) and macular layer thickness (96 studies); 69 studies looked at both of these measures. For 25 (18.7%) of the papers, no mention of image quality criteria or exclusion of subjects or eyes because of image quality criteria was found. For another 17 (12.7%) of the papers, there was a statement about the exclusion of subjects or eyes from analysis because of image quality, but no specific criteria were stated. Twenty-five papers used OCT angiography to report a variety of parameters including: vessel density, perfusion density, foveal avascular zone size, capillary perfusion index, and optic nerve flow intensity. Twenty-one used measurements generated by the device, while 4 used a custom image processing algorithm in either Matlab or ImageJ. The vessel density or perfusion density was typically calculated as the area of vessels (or perfusion) divided by the total image area, and they were typically reported as a ratio or percent. For vessel density, some studies calculated the length of vessels divided by the image area,^27^ and some studies excluded the area of the foveal avascular zone.^28^

One hundred seventeen (87.3%) studies performed bilateral eye imaging (Table 2), but only 89 (66.4%) studies performed bilateral image analysis (Table 3). Among the 42 studies that analyzed 1 eye per subject, the most common method was random selection (22 studies, 52.4%). Among the 89 studies that had bilateral eye image analysis performed, 53 (59.6%) had analysis at the eye level by combining data from the left and right eye so that each eye (rather than each subject) was the unit of analysis. The remaining studies used a variety of approaches, including using the mean of 2 eyes (18 studies, 20.2%), intereye difference of affected and unaffected eyes (16 studies, 18.0%), or separate analysis for left eyes and right eyes (10 studies, 11.2%) (Table 3). Among the 53 studies that performed analysis at the eye level for data from both eyes, the intereye correlation was adjusted in 38 (71.7%) studies, while 15 (28.3%) studies did not account for the intereye correlation in the bilateral analysis. Among those 38 studies that performed intereye correlation adjustment in bilateral eye level analysis, 21 (55.3%) used generalized estimating equations and 13 (34.2%) used mixed-effects models (Table 3). Additionally, among the 53 studies that had bilateral eye level analysis, a greater percentage of papers published in neurology journals adjusted for intereye correlation (23 of 26 neurology journals [88.5%] vs. 14 of 27 ophthalmology journals [51.9%], *P* = 0.004). Further, a greater percentage of papers with corresponding authors that were neurologists adjusted for intereye correlation (17 of 20 neurologists [85.0%] vs. 14 of 26 ophthalmologists [53.9%], *P* = 0.046) (Table 4).

**Table 4:** Factors Associated with Adjustment for Intereye Correlation among Studies That Performed Data Analysis from Both Eyes (N = 53)

Factor	Number of Studies	Adjusted for Intereye Correlation (Row %)	Chi-Square *P* Value
Type of journal paper published			0.004
Clinical neurology	26	23 (88.46)	
Ophthalmology	27	14 (51.85)	
Year of publication			0.52
2013–2018	23	15 (65.22)	
2019–2023	30	22 (73.33)	
Specialty of corresponding author			0.046
Ophthalmology	26	14 (53.85)	
Neurology	20	17 (85.00)	
Other	7	6 (85.71)	
Country of corresponding author			0.14
United States	28	22 (78.57)	
Not United States	25	15 (60.00)	
Disease of interest			0.13
Multiple sclerosis	28	22 (78.57)	
Alzheimer disease	14	10 (71.43)	
Parkinson disease	10	4 (40)	
Other	1	1 (100.00)	
OCT study type			0.47
Cross-sectional	38	26 (68.42)	
Longitudinal	14	11 (78.57)	

The sample size among analyzed papers varied widely and is defined as the total number of eyes analyzed, including both case and control eyes. The median was 166 eyes (range 7–144 166). After excluding 7 papers with >1000 eyes, the median sample size was 150 (range 7–195) eyes.

Further analysis of statistical methodologies in the studies demonstrated that 37 (27.6%) studies corrected for multiple comparisons given multiple OCT measurements that were evaluated (Table 3). Only 15 (11.2%) studies reported statistical power calculations.

## Discussion

OCT measurements have potential as a noninvasive modality for assessing neurodegeneration, and additional studies are expected in this growing research field. When evaluating any clinical diagnostic tool, robust study design and statistical analysis are required to make strong conclusions. To the best of our knowledge, this is the first paper to investigate the methodology characteristics of the published literature on the relationship between OCT measurements and neurodegenerative diseases.

The neurodegenerative diseases evaluated by these studies have the potential to involve both eyes. The measures taken from 2 eyes of the same patient are usually positively correlated, while the measures from 2 eyes from 2 different subjects (eg, each subject only contributes 1 eye) are independent. The statistical analysis cannot analyze the data from 2 eyes of the same subject (eg, correlated) in the same way as data from 2 eyes from 2 different subjects (eg, independent). Importantly, the results of this study demonstrate that a majority (87%) of investigations performed bilateral imaging, but a notable percentage of studies (31%) only analyzed data from 1 eye, potentially leading to an inefficient analysis by not utilizing the available data from the second eye. Furthermore, among all studies that performed bilateral analysis at the eye level, more than one quarter (28.3%) did not adjust for the intereye correlation in the statistical analysis. If a study uses both eyes from subjects with each eye as the unit of analysis, then ignoring the intereye correlation (as if they were independent) in the statistical analysis can cause the *P* value to be too small, and the 95% confidence interval to be too narrow, when 2 eyes of the same subject are in the same comparison group.^23^^,^^24^ In assessing the current literature, the results of this investigation are 2 sided. While a significant proportion of studies performed bilateral imaging analysis and accounted for intereye correlation, there is still a significant portion of studies that did not. When appropriate for a study, we believe the optimal statistical methodology would utilize data from both eyes with adjustment of the intereye correlation.^23^^,^^24^ It is of further interest that this study found that among studies with bilateral eye data, a greater proportion of studies from neurology rather than ophthalmology journals adjusted for intereye correlation. Similarly, a greater proportion of corresponding authors of publications that adjusted for intereye correlation were neurologists rather than ophthalmologists. The reasons for a greater proportion of neurology journal and neurologists are unclear. It also should be noted that ophthalmologists were sometimes the corresponding author in a neurology journal publication.

Regarding statistical methodology, it is also notable that only 28% corrected for multiple comparisons. Especially for studies evaluating each of the retinal layers of the macula, it is not uncommon for multiple retinal layer associations to be tested. The number of comparisons can be further increased in a study if each of the different sectors of a topographical map, such as the ETDRS grid, is tested for an association with the disease of interest. An unadjusted *P* value in a study testing many hypotheses for various OCT measurements can lead to false statistical significance, especially if the finding is not consistent with a prespecified hypothesis.

Excellent image quality of OCT images is critical for reliable assessment of OCT measurements. We could not identify a statement about OCT image quality criteria in 19% of studies, and another 13% of the studies did apply image quality criteria but did not specifically define the quality criteria to determine which images were included in the analysis. While readers might assume that most studies would exclude poor quality images from analysis, a specific statement about the quality criteria can be considered a characteristic of a rigorously designed study. Among other methodology variables, this limits the ability to compare data across studies and would be needed to combine data sets optimally. The obvious problems (O), poor signal strength (S), centration of scan (C), algorithm failure (A), retinal pathology other than multiple sclerosis related (R), illumination (I), and beam placement (B) (OSCAR-IB) criteria, which was published in 2012 and utilized by 28%, represents an effort to standardize image quality control criteria.^29^ Similarly, the more recent Advised Protocol for OCT Study Terminology and Elements (APOSTEL2) criteria is an effort to standardize reporting from these types of studies.^30^

Regarding study design, we found that 77% were cross-sectional and 23% were longitudinal. Of the 31 studies that were longitudinal, 25 (81%) were studies of multiple sclerosis. Conditions such as Alzheimer disease and other dementias can be difficult to perform longitudinally because the disease progression may limit patient follow-up or the ability to obtain adequate quality images. Nevertheless, more longitudinal dementia studies are needed, as they can be valuable to understand how these OCT measurements relate to disease progression. Longitudinal studies could be facilitated by enrolling patients with mild disease severity at baseline and following the disease characteristics and ocular measurements over time.

Diagnostic criteria for neurodegenerative diseases continue to evolve, and it should be noted that autopsy is the gold standard for the diagnosis of Alzheimer disease and other dementias. While magnetic resonance imaging is highly sensitive for multiple sclerosis,^31^ autopsy is also needed to have full diagnostic certainty for Parkinson disease.^32^ Especially for dementia, standard of care clinical criteria for diagnosis can have variable accuracy, and cases within studies may not reflect the potential for >1 concurrent neurologic diagnosis that could affect the retina.^33^, ^34^, ^35^ For example, studies with autopsy data have found that the clinical diagnosis of Alzheimer disease without biomarkers is inaccurate for ≥25% of cases.^33^^,^^36^ As only a small percent of Alzheimer disease cases are familial, the use of CSF and PET biomarkers for Alzheimer disease in clinical studies deserves evaluation as these biomarkers have >90% sensitivity and specificity.^37^^,^^38^ Of the 42 Alzheimer disease studies, we found that only 8 (19%) utilized CSF biomarkers and 11 (26%) utilized PET imaging. Many studies of clinically diagnosed Alzheimer disease have found thinning of the retinal nerve fiber layer and ganglion cell layer,^11^ but a systematic review and meta-analysis of only those OCT studies of Alzheimer disease utilizing biomarkers for disease confirmation found only weak evidence for thinning of the retinal nerve fiber layer and no significant findings for the ganglion cell layer.^39^ The use of biomarkers or lack thereof for diagnosis confirmation should be considered when interpreting dementia studies. With the US Food and Drug Administration approval of CSF and PET biomarkers for Alzheimer disease in recent years, as well as the ongoing development of plasma biomarkers,^33^^,^^40^ the utilization of biomarkers in clinical studies will likely increase.

Limitations of this study include its retrospective nature and dependence on published methodology and results regarding each investigated manuscript. Further, our manuscript selection criteria were meant to capture papers from top journals for which good quality in study design is expected. This could limit the generalizability of our findings to all studies in this field. Nevertheless, our study had a large sample size of 134 papers and is likely representative of papers from recognized journals in this research field. Finally, we acknowledge that multiple sclerosis has some clinically distinct features compared to neurodegenerative conditions such as Alzheimer disease and Parkinson disease, and the field of OCT research for multiple sclerosis can be considered more developed. Still, we believe it is reasonable to investigate this group of known neurologic conditions together to evaluate the approaches to studies of OCT measurements during this 10-year period.

This unique investigation of papers in this growing field aims to inform researchers of recent publication trends and highlight areas for improvement in study design and statistical analysis. An optimal study design would maximize the use of the acquired eye data and explicitly utilize image quality criteria. For future studies of dementia, it is worthwhile to consider the use of biomarkers for Alzheimer disease if autopsy data are not available. It is also expected that future methodologies will utilize artificial intelligence, and this will also have its own issues with methodology that will need to be refined. OCT measurements are a promising prognostic and diagnostic tool in neurodegenerative disorders, though their utility can only be as strong as the study design and statistical methodology underlying them.
