# Supplementary material for: Characteristics of Study Design and Statistical Analysis in OCT-Based Studies of Neurodegeneration
Source: Ophthalmol Sci. 2025 Oct 8;6(1):100961. doi: 10.1016/j.xops.2025.100961 (PMC12689194; doi:10.1016/j.xops.2025.100961)
Supplement: Supplemental Table 1 [file mmc1.pdf]

Supplemental Table 1.

Scan Protocols (Total N = 134)	(N)	(%)
**OCT**		
Optic Disc/Optic Nerve Head		
12° high-speed peripapillary scan	1	0.75
3.5 mm diameter optic nerve head scan (2048 A-scans)	1	0.75
4.5×4.5mm peripapillary scan	1	0.75
6 mm ring scan	1	0.75
BMO-, APS-centered scans	2	1.49
RNFL-N (Spectralis); NR for Cirrus	1	0.75
RNFL-N protocol, 30 × 25° macular scan (61 vertical scans, 768 A scans per s, automatic real time (ART) = 15)	1	0.75
RT-vue XR Avanti ONH analysis	1	0.75
RTVue optic nerve head map protocol	1	0.75
Spectralis 3.5-mm standard circle scan protocol	1	0.75
Spectralis N-Site Axonal	1	0.75
Spectralis RNFL protocol	1	0.75
Spectralis RNFL-N protocol	1	0.75
Stratus fast RNFL thickness (3.4) scan	1	0.75
fast RNFL protocol	1	0.75
optic disc 6.0 × 6.0 mm three-dimensional scan	1	0.75
peripapillary 12º ring scan (1536 A-scans, 16ART100)	1	0.75
peripapillary 4.5 × 4.5-mm2 scans	1	0.75
3.4 mm optic disc scan	1	0.75
Macula Scan		
20 × 15° raster scans	1	0.75
20° macular volume scans (25 raster scans, ART 25)	1	0.75
3D macular volume scan	1	0.75
3×3mm2, 6×6mm2 macula scans	1	0.75
3×3×3 mm volumetric scan	1	0.75
512 × 128 macular scan	1	0.75
6x6 mm macular scan	1	0.75
6×6×1.68 mm 512×512×480 voxels scans; 6×6×2.30 mm 512×512×885 voxels scans	1	0.75
7 × 7 × 2 mm, 75 b-scans, 743 a-scans per b-scan	1	0.75
9 × 9 mm macula scan (256 serial parallel B-scans)	1	0.75
Cirrus 512 × 128 scan protocol;	1	0.75
Optovue radial scanning (18 lines);	1	0.75
RTVue enhanced depth imaging protocol	1	0.75
Spectralis macula scan	1	0.75
Spectralis macular volume protocol; RTVue-100 MM6 protocol	1	0.75
Spectralis posterior pole analysis, enhanced depth imaging	1	0.75
Topcon 3-dimensional macular volume scan	2	1.49
enhanced depth imaging	1	0.75
high-resolution macular volume scan	1	0.75
Macula / Fovea		
21-line EDI foveal scan	1	0.75
30° × 25° fovea scan (31 B-scans)	1	0.75

3×3mm, 6×6mm fovea images	1	0.75
3×3mm2 fovea scan	1	0.75
6 × 6 mm fovea scan (512 A-scans (per B-scan) × 128 B-scans)	1	0.75
6×6 mm fovea scan (128 B-scans, 512 A-scans per B, 2 mm depth)	1	0.75
fovea scans (512 A-scans per line)	1	0.75
Optic Disc/Peripapillary and Macula / Fovea		
12° 100 ART peripapillary; 30° x 15° 25 ART perifoveal	1	0.75
peripapillary 12º diameter scan (ART: 100; 1,536 A-Scans per B-scan); 20x20 degree horizontal raster fovea scan (25 B-scans, ART 9; 512 A-Scans per B-scan)	1	0.75
peripapillary 4.5 × 4.5-mm2 scans	1	0.75
peripapillary 3.5 mm ring scan (thickness); fovea raster scan matrix size 20 x 20; 25 sections of 240m; 6 mm ring area (volume)	1	0.75
peripapillary 3.5 mm ring scan (12°, 768 A-scans); 20°×20° macular volume scan (25 B-scans, vertical alignment, ART 49, density 240 m, 4.7 scans/s)	1	0.75
Optic Disc/Peripapillary and Macula		
12° peripapillary ring scan; 20 × 20-degree horizontal raster scan	1	0.75
20° × 20° macular scan (49 B-scans); axonal optic nerve ring scan (ART 100)	1	0.75
12° 3.4mm peripapillary scan 50 ART; macular scan 25ART 25 vertical scans	2	1.49
20°×20° macular area scan (25 horizontal axial scans, 512 A-scans per B-scan, 49 frames per B-scan), peripapillary 12° diameter ring scan (768 A-scans per B-scan, 100 frames per B-scan)	1	0.75
25°x30° macula scan (61 vertical B-scans, high-speed mode, automatic real time (ART) 12-15); peripapillary 3.4 mm ring scan (ART 16-100)	1	0.75
3.4-mm (12°) ONH ring scan (1536 A scans, automatic real-time tracking [ART]: 100 averaged frames); macular volume scan (20° × 20 °, 512 A scans, 25 B scans, vertical alignment, ART: 16 averaged frames)	1	0.75
30°×25° macular scan (61 vertical B-scans, ART 13); peripapillary 3.4 mm ring scan (ART 100)	1	0.75
3×3mm macula map, 2.4×4mm disc map	1	0.75
6 mm, 30° macular scan; peripapillary 3.5 mm ring scan	1	0.75
6 x 6 mm macular scan, peripapillary 3.5 mm ring scan	1	0.75
6 × 6 mm2 macula scans, 4.5 × 4.5 mm2 peripapillary scans	1	0.75
8×8mm macular scan; 8×8mm optic nerve head scan; 3×3×3mm3 optic nerve head 3D volumetric scan	1	0.75
9 x 9 mm macula scan (512 x 128 scan density), 6 x 6 mm optic disc scan (512 x 128 scan density)	1	0.75
Cirrus ONH Cube 200×200 scan, Cirrus automated macular volume cube 200×200 or 512×128 scan, Spectralis 6x6 macular volume scan	1	0.75
Cirrus Optic Disc Cube 200 × 200 protocol, Cirrus Macular Cube 512 × 128 protocol	5	3.73
Cirrus glaucoma RNFL protocol; Cirrus macular cube	1	0.75
Cirrus macular cube 200 × 200, Cirrus optic disc cube 200 × 200, Spectralis fast retinal scan, Spectralis RNFL with TruTrack	1	0.75
Fast macular protocol, optic disc protocol, Macular Cube 200×200 protocol	1	0.75
Optic Disc Cube 200 × 200, Macular Cube 512 × 128	9	6.72
Spectralis Fast Retinal protocol, glaucoma RNFL protocol, Nsite Axonal Analytics RNFL-N protocol, Cirrus Macular Cube 200 × 200	1	0.75
Spectralis axonal RNFL, radial macular protocol	1	0.75
Spectralis fast macular cube; RNFL-N Axonal Analytics	2	1.49
fast macular protocol, RNFL protocol	1	0.75
macula radial pattern protocol, peripapillary circular scan	1	0.75
macular cube 512 × 128, optic disc 200 × 200	2	1.49

optic disc volume scan (15°×15°, 37 B-scans), peripapillary ring scan (12°, 1 B-scan), macular volume scan (20°×20°, 49 B-scans)	1	0.75
peripapillary 12º ring scan, macular scans	1	0.75
peripapillary 3.4 mm ring scan (100 A scans), horizontal macula volume scans	1	0.75
peripapillary 3.4 mm ring scan (ART 100); 30° × 25° macular area scan (61 vertical B-scans, 768 A-scans per B-scan)	1	0.75
peripapillary 3.4 mm ring scan (ART 50); macula scan (25 vertical scans, 25 ART)	1	0.75
peripapillary 3.4 mm ring scan, ONH Cube 200×200 scan, 200×200 or 512×128 macular volume cube	1	0.75
peripapillary 3.5 mm ring scan (12°, 768 A-scans); 20°×20° macular volume scan (25 B-scans, vertical alignment, ART 49, density 240 m, 4.7 scans/s)	1	0.75
peripapillary 3.5 mm ring scan (thickness); fovea raster scan matrix size 20 x 20; 25 sections of 240m; 6 mm ring area (volume)	1	0.75
peripapillary RNFL, macular ETDRS and macular multilayer	1	0.75
peripapillary ring scan (12°); 20°×20° macular volume scan (25 B-scans, vertical alignment)	2	1.49
peripapillary ring scan (12°); 30°× 30° macular volume scan (6 B-scans)	1	0.75
peripapillary ring scan (12°, 1536 A-scans, 1 B-scan, no predetermined automatic real time (ART)); 20°×20° macular volume scan (512 A-scans, 49 B-scans, vertical alignment, ART 16)	1	0.75
**OCTA**		
3x3mm Macula		
3 mm × 3 mm macular OCTA scan, 6 mm × 6 mm macula scan	1	0.75
3 × 3mm OCTA scan (304 x 304 A- * B-scans);	1	0.75
3×3mm2 OCTA scan (245×245, 4 B-scans)	1	0.75
6x6mm Macula		
6 x 6 mm OCTA	1	0.75
6 x 6 mm OCTA scan	1	0.75
6 mm×6 mm OCTA scan (512×512 pixels, 512 A-scans, 4 B-scans)	1	0.75
6 × 6 mm2 (304 × 304 pixel) fovea OCTA scan	1	0.75
6×6mm OCTA scan, enhanced deep imaging	1	0.75
Other		
4.5x4.5 ONH	1	0.75
**OCT Optic Disc and/or Macula And OCTA**		
200 × 200 optic disc cube scan, peripapillary 4.5 × 4.5-mm OCTA scan, macular 6 × 6-mm OCTA scan	1	0.75
3 × 3-mm, 6 × 6-mm fovea OCTA scans; 512 × 128 macular cube; 200 × 200 optic disc cube	1	0.75
512 × 128 macular cube scan, 200 × 200 optic disk cube scan, 21-line enhanced depth imaging scan, 3 x 3 mm OCTA scan	1	0.75
512 × 128 macular scan; 3 × 3 mm2 OCTA scan	1	0.75
6 x 6 mm OCTA scan, 512×128-m macular cube, 200×200-m optic disc cube, 21-line EDI foveal scan	1	0.75
Triton 3D Wide protocol; Triton SS-OCT-Angio™	1	0.75
**Not Recorded**	14	10.45

Abbreviations: ART = automatic real time; BMO = Bruch's membrane opening; EDI = enhanced depth imaging; OCT = optical coherence tomography; OCTA = optical

coherence tomography angiography; ONH = optic nerve head; RNFL = retinal nerve fiber layer; SS = swept source.
